# The Expression and Clinical Significance of PCNAP1 in Hepatocellular Carcinoma Patients

**DOI:** 10.1155/2022/1817694

**Published:** 2022-02-18

**Authors:** Yanhong Chen, Jiao Zhang, Jia Liu, Jianguo Wang, Chao Shi, Lu Lu, Xing Cheng, Guoping Niu, Shuangshuang Zhang

**Affiliations:** Department of Clinical Laboratory, Xuzhou Central Hospital, China

## Abstract

**Background:**

Long noncoding RNAs (lncRNAs) play an important role in many cancer progression. The aim of this study was to evaluate the expression level and clinical significance of the lncRNA, proliferating cell nuclear antigen pseudogene 1 (PCNAP1), in cancer tissue and the plasma of patients with hepatocellular carcinoma (HCC).

**Methods:**

Quantitative real-time polymerase chain reaction was used to detect the expression of PCNAP1 in HCC tissue, adjacent tissue, and plasma. Spearman's rank correlation analysis was performed to assess relationships among cancer tissue, plasma PCNAP1, and plasma AFP. Kaplan–Meier analysis was used to assess survival of HCC patient with high and low expression of PCNAP1. The survival difference was compared by the log-rank test. The use of plasma levels PCNAP1 for diagnosing HCC was evaluated by receiver operating characteristic curve analysis.

**Results:**

The expression of PCNAP1 in HCC tissue was significantly higher than in adjacent tissue (*P* < 0.01). The PCNAP1 levels were related to the TNM stage, lymph node metastasis, and tumor maximum diameter (*P* < 0.05) but were not related to gender and age (*P* = 0.459 and 0.656). Patients with greater levels of PCNAP1 had poorer survival than patients with lower levels of expression (*P* < 0.01). Compared to the healthy control group, a gastric cancer group, and a colorectal cancer group, HCC patient plasma levels of PCNAP1 were significantly greater (*P* < 0.01). The area under the curve (AUC) of plasma PCNAP1 in HCC patients was 0.83 (95% CI: 0.78-0.88). With a cut-off value of plasma PCNAP1 at 1.27, an HCC diagnostic sensitivity of 70.08%, and a specificity of 85.04%, was the maximum diagnostic efficiency achieved.

**Conclusion:**

This study demonstrates PCNAP1 levels to be increased in HCC patients. As such, PCNAP1 may be a new tool useful in disease diagnosis and prognosis.

## 1. Introduction

Hepatocellular carcinoma (HCC) is a primary cancer of the liver and the third leading cause of cancer-related death [1]. HCC is one of the most common gastrointestinal tumors. Worldwide, nearly 700,000 people die per year from HCC, with mortality increasing yearly [2]. The onset of HCC is delitescent in nature, with no clinical specificity. When clinically diagnosed, most patients have progressed to the middle or late stages of the disease [3]. For the last few decades, investigations have focused on the diagnosis and effective treatment of HCC. Although significant progress has been made, radical treatment methods such as surgery and liver transplantation have not significantly improved the overall survival rate for HCC patients [4, 5]. Therefore, there is an urgent need to develop effective early diagnostic markers and therapeutic targets to improve early diagnosis and the overall survival rate of HCC patients. lncRNAs are a class of endogenous RNA molecules longer than 200 nucleotides in length. Although lncRNAs have no protein-coding capacity, they are important epigenetic regulators of cellular metabolism [6], proliferation [7, 8], and apoptosis [9]. A variety of diseases are closely related to the abnormal expression or dysfunction of lncRNAs [10]. Further, lncRNAs are involved in cell cycle progression, chromatin modification, and transcriptional activation, as well as tumor formation, invasion, and metastasis [11, 12]. For example, the lncRNA, Ptn-dt, promotes the proliferation of HCC cancer cells by interacting with the HuR protein [13]. lnc-GALH enhances the metastasis of HCC by a similar mechanism [14]. With the development of RNA sequencing technology, many lncRNAs have been found to play important roles in HCC, although the precise function of these lncRNAs remains unclear. Feng et al. [15] found that lncRNA PCNAP1 can enhance HBV replication and hepatocarcinogenesis. They also demonstrated the host lncRNA, proliferating cell nuclear antigen pseudogene 1 (PCNAP1), and enhanced HBV replication through modulation of miR-154/PCNA/HBV cccDNA signaling, which is the PCNAP1/PCNA signal that drives hepatocarcinogenesis. The aims of this study were to explore the expression levels and clinical significance of PCNAP1 in HCC patients. We found the expression of PCNAP1 to be at relatively high and stable levels in cancer tissue and the plasma of HCC patients, and that PCNAP1 levels correlated with alpha-fetoprotein (AFP), a clinical tumor marker of HCC. Moreover, levels of PCNAP1 were found to distinguish HCC from other gastrointestinal tumors.

## 2. Materials and Methods

### 2.1. Patients and Samples

This study was conducted in Xuzhou Central Hospital and was approved by the Research Ethics Committee of Xuzhou Central Hospital. A total of 89 patients (59 males and 30 females), diagnosed with HCC, were recruited from Xuzhou Central Hospital, between September 2017 and June 2020. The mean age was 58 ± 16 years. Paired tumor and adjacent tissues (>2 cm distance from cancer tissue) were preserved at the same time. These tissues were collected within 15 min and stored in liquid nitrogen for reverse transcription-quantitative polymerase chain reaction (RT-qPCR). A total of 127 HCC nonsurgical patients (86 males and 41 females) with HCC were also recruited with an average age of 59 ± 18 years. As well, 100 gastric cancer patients (78 males and 22 females, 56 ± 17 years) and 100 colorectal cancer patients (64 males and 36 females, 54 ± 18 years) were recruited as disease control groups. Another 127 healthy subjects (78 males and 49 females, 58 ± 18 years) were recruited as the healthy control group (HC). The plasma of patients in each group was collected and stored at -80°C. The AFP concentration in plasma of patients with HCC was assessed by radioimmunoassay. All patients with one of the following conditions were excluded from the study: (1) extrahepatic malignant tumors and/or a noninvasive liver, (2) hepatitis or liver cirrhosis caused by other diseases, and (3) patients with other diseases affecting the results of the study. All selected patients had no other medical history and did not receive radiotherapy, chemotherapy, or immunotherapy. All patients signed informed consent documents before investigation and sampling.

### 2.2. RNA Extraction

Total cells from tissue samples or plasma were collected and resuspended in lysis buffer (10 mM NaCl, 20 mM MgCl, 10 mM Tris-HCl, pH 7.8, 5 mM DTT, and 0.5% NP-40) and kept in ice for 5 minutes. Treated samples were subjected to protease treatment for 20 minutes at 37°C by adding an equal volume of proteinase K solution (300 mM NaCl, 0.2 M Tris-HCl, pH 7.5, 25 mM EDTA, 2% SDS, and 0.1 mg/ml proteinase K). RNA was purified using the QIAzol Lysis Reagent (Qiagen, Germany) followed by DNase treatment. cDNA was synthesized using the QuantiTect Reverse Transcription Kit (Qiagen, Germany) according to the manufacturer's protocol.

### 2.3. qRT-PCR Analysis

qRT-PCR was performed using a Bio-Rad MyCycle (Bio-Rad, CA, USA) according to the manufacturer's instructions. *β*-Actin was used as a reference gene. The PCR primer for PCNAP1 (Gene ID: 359806) was F: 5′-UCUTGGCTTATTAGTCGTAGCTA-3′, R: 5′-AATATGTGTGTCGCGGGATG-3′; the PCR primer for *β*-actin was F: 5′-AATATGTGTGTCGCGGGATG-3′, R: 5′- CTCCTTAATGTCACGCACGCACGA-3′. The reaction conditions were 95°C, 15 s; 60°C, 30 s; 74°C, 30 s; and 72°C, 20 s, with 40 cycles of amplification. All experiments were repeated at least three times. The expression of PCNAP1 was evaluated using a comparative cycle threshold (CT).

### 2.4. Statistical Analysis

Statistical analysis was conducted using GraphPad Prism 8.0. All data are presented as means ± SD. Differences between two groups were analyzed by *t*-test, while those among three groups were assessed via one-way ANOVA. Spearman's rank correlation analysis was used to analyze the relative expression levels of PCNAP1 and AFP in plasma and in HCC tissues. The HCC diagnostic value of plasma PCNAP1 was estimated by receiver operating characteristic curve analysis (ROC). *P* < 0.05 was considered to be statistically significant.

## 3. Results

### 3.1. Relationships among PCNAP1 Expression, Clinicopathological Characteristics, and Overall Survival

**Figure 1:**
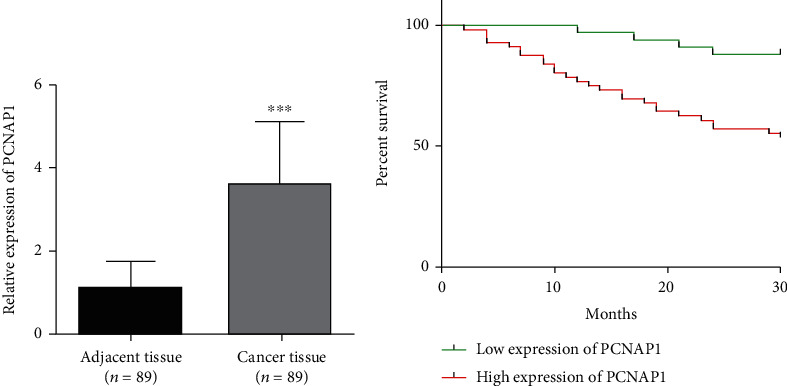
(a) The relative levels of PCNAP1 were examined by RT-qPCR in HCC cancer tissue and adjacent tissue. (b) The relationship between PCNAP1 expression and survival by Kaplan–Meier analysis.

The expression levels of PCNAP1 in tumor and adjacent tissue were assessed by qRT-PCR assay. As shown in Figure 1(a), PCNAP1 expression in HCC cancer tissue (3.59 ± 1.55) was significantly higher than that in adjacent tissue (1.09 ± 0.66, *t* = 14.01, *P* < 0.01). In order to explore clinical significance, relationships between PCNAP1 expression and HCC clinical characteristics were assessed. The 89 patients with HCC were divided into high- and low-expression groups based on the cut-off value derived from an ROC curve. There were 56 cases in the high expression group and 33 in the low expression group. Kaplan–Meier analysis demonstrated overall survival of patients in the high PCNAP1 expression group to be shorter than in the low expression group (*χ*^2^ = 10.86, *P* < 0.01) (Figure 1(b)). Further, HCC expression of PCNAP1 was related to TNM clinical stage, lymph node metastasis, and maximum tumor diameter (*χ*^2^ = 10.337, 6.718, and 8.164; *P* < 0.05), but not related to gender or age (*χ*2 = 6.228 and 5.309; *P* = 0.459 and 0.656) (Table 1).

### 3.2. Plasma PCNAP1 Levels in Patients with HCC, HC, Other Disease, and Control Groups

**Figure 2:**
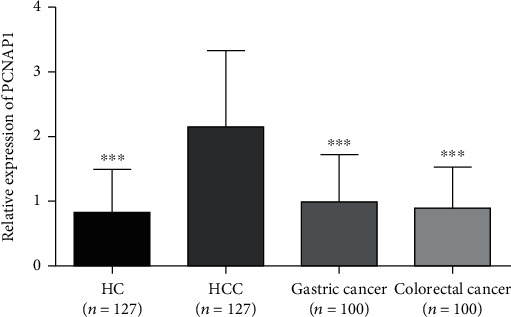
Relative levels of PCNAP1 in plasma of HCC patients, healthy controls, gastric cancer, colorectal cancer, and disease subjects were examined by RT-qPCR.

The plasma levels of PCNAP1 in patients with HCC, HC, gastric cancer, and colorectal cancer were 2.15 ± 1.20, 0.82 ± 0.67, 1.00 ± 0.73, and 0.89 ± 0.63, respectively. The level of PCNAP1 in HCC plasma was significantly higher than in other groups (*t* values were 10.81, 8.39, and 9.49, respectively; *P* < 0.01) (Figure 2). Compared to the HC group, the level of PCNAP1 in gastric cancer and colorectal cancer groups was not significantly increased and was not statistically significant (the *t* values were 1.90 and 0.74, respectively; the *P* values were 0.06 and 0.46, respectively). These results indicate that plasma PCNAP1 levels distinguish HCC patients from other cancers and may be a new plasma marker for HCC.

### 3.3. Relationships between PCNAP1 in HCC Plasma with Cancer Tissue and Plasma AFP

**Figure 3:**
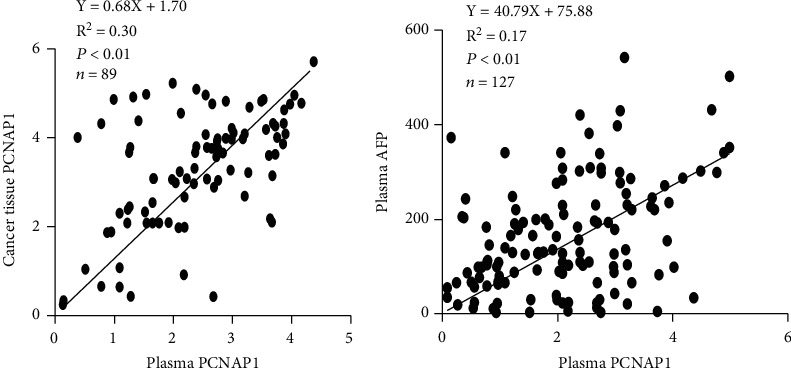
Spearman's rank correlation analysis was taken for analysis of the relationship between tissue and plasma PCNAP1 and (a) cancer tissue and (b) plasma AFP in HCC patients.

We then assessed the potential use of PCNAP1 as a marker for HCC, similar to the common use of AFP. Spearman's rank correlation analysis showed that the level of PCNAP1 in plasma of HCC patients was positively related to the expression of PCNAP1 in cancer tissue (*r* = 0.54, *P* < 0.01) (Figure 3(a)). Further, the level of PCNAP1 in plasma of HCC patients was positively related to AFP (141.0 ± 113.1) (*r* = 0.41, *P* < 0.01) (Figure 3(b)).

### 3.4. Diagnostic Value of PCNAP1 in HCC Patients

**Figure 4:**
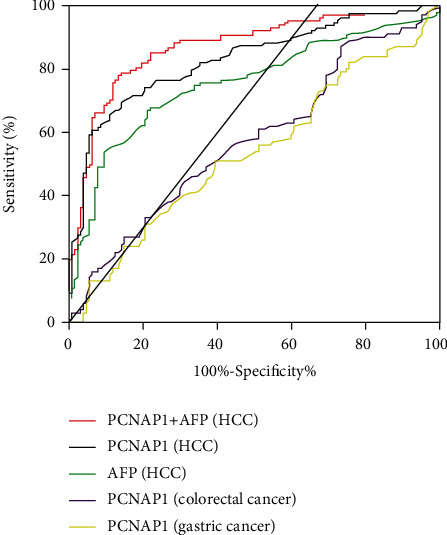
ROC curve analysis of the use of plasma PCNAP1 for diagnosis and differential diagnosis of hepatocellular carcinoma.

To explore the diagnostic value of PCNAP1, an ROC curve was constructed. The outcome showed an AUC of 0.83 (95% confidence interval (CI): 0.78–0.88) with a sensitivity of 70.08% and a specificity of 85.04%. The ideal cutoff value was 1.27. When plasma PCNAP1 and AFP values were combined for the diagnosis of HCC, the AUC increased to 0.87 (95% CI: 0.82-0.92), and the sensitivity and specificity were 0.79 and 0.86, respectively. Interestingly, the AUC for chronic gastric cancer and colorectal cancer were 0.57 and 0.54, indicating that PCNAP1 was significantly more advantageous for the diagnosis of HCC (Figure 4 and Table 2).

## 4. Discussion

Hepatocellular carcinoma (HCC) is a severe, life-threatening malignancy. Pathogenesis is complicated, and there are many related genes. The disease is multifactorial and a consequence of a multigene, multistep regulatory process [15, 16]. Since the 1970s, serum AFP (alpha-fetoprotein) levels have been used as a clinical biomarker of HCC [17–19]. Unfortunately, when 70%–90% of HCC patients are AFP positive, the specificity is 72%–90%, but the sensitivity is only 9%–32% [20]. Further, approximately one-third of HCC patients have normal AFP levels [21]. As such, AFP does not provide necessary clinical specificity or sensitivity, which results in misdiagnosed HCC. Therefore, an HCC diagnostic marker with greater specificity and sensitivity is essential for discrimination of HCC from other malignant tumors. Such a marker could be used to diagnose HCC at an early stage, permitting more effective early treatment for HCC.

Long noncoding RNAs (lncRNAs) are a class of RNA molecules (excluding rRNA) that do not encode proteins, with a length of more than 200 bp. Tens of thousands of lncRNAs exist that specifically bind to the complementary sequences of target genes, thereby promoting or inhibiting protein synthesis [22]. Each lncRNA has multiple target genes, and several lncRNAs can regulate the same gene. Further, the expression of multiple genes can be achieved through one lncRNA. The effects of that lncRNA can be refined by combination with several other lncRNAs. It has been reported that approximately 40%–95% of human genes are influenced by lncRNAs [23–26]. *In vivo*, lncRNAs are involved in the development, maturation, proliferation, differentiation, and apoptosis of a variety of cells [27]. As such, lncRNAs are newly discovered, potentially powerful *in vivo* regulatory factors. lncRNAs play a very important role in the development of multiple physiological systems. The abnormal expression of lncRNAs can disrupt normal physiological function resulting in a variety of diseases. In the last decade, studies demonstrated lncRNAs to be directly involved in tumorigenesis and progression of known tumors [28, 29]. lncRNA, PCNAP1 (proliferating cell nuclear antigen pseudogene 1), and its ancestor PCNA, a DNA polymerase, coordinate and maintain the integrity of the genome at the genetic and epigenetic level, participating in DNA replication and repair by interaction with many partner proteins [30]. PCNA, as a marker of cell proliferation, can be used to grade different tumors. PCNA has at least four effective pseudogenes (PCNAP1, PCNAP2, PCNAP3, and PCNAP4) [31]. Feng et al. found that lncRNA PCNAP1 can enhance HBV replication and hepatocarcinogenesis [32], but the function and/or mechanism of PCNAP1 in HCC is also unclear, although complex interactions with multiple factors and complex environments are known to regulate PCNAP1 expression [33].

This study demonstrated PCNAP1 in the cancer tissue and plasma of HCC patients. Further, cancer tissue PCNAP1 was shown to relate to clinical symptoms of HCC patients. Plasma PCNAP1 may have a clinical diagnostic value for HCC patients; in that compared to other gastrointestinal tumors (gastric cancer and colorectal), the level of PCNAP1 was found to be greater (*P* < 0.05). Further, PCNAP1 levels in liver cancer tissue were significantly greater than in adjacent tissue (*P* < 0.01), suggesting that PCNAP1 may be a new biomarker for HCC diagnosis. There was a positive relationship between PCNAP1 and AFP in the plasma of HCC patients (*r* = 0.41, *P* < 0.01), and the level of PCNAP1 in plasma was also positively related to PCNAP1 in cancer tissue (*r* = 0.54, *P* < 0.01). Further, PCNAP1 in cancer tissue of HCC patients was related to TNM staging, lymph node metastasis, and maximum tumor diameter. No relationship to age or gender was found. The AUC for plasma PCNAP1 in HCC patients was 0.83 (95% CI: 0.78-0.88). With a cut-off value of PCNAP1 at 1.27, its diagnostic sensitivity and specificity were 70.08% and 85.04%, respectively, with maximum diagnosis achieved at this level. When plasma PCNAP1 and AFP were combined for HCC diagnosis, the AUC was 0.87 (95% CI: 0.82-0.92). There are shortcomings to this study. First, potential confounders such as the screening of clinical cases and differences in treatment may have affected the results. Second, this study was limited to the cases screened by the hospital, which may not represent the local area or even the national caseload. Third, the study was cross-sectional. Further, it is necessary to confirm the role of PCNAP1 in HCC, as well as in cancer cell differentiation, infiltration, metastasis, clinical treatment, and prognosis. Those issues will be addressed in our future studies.

This study analyzed the expression of PCNAP1 in the cancer tissue and plasma of HCC patients and found relationships among PCNAP1 and patient clinicopathological characteristics. As well, potential clinical diagnostic value for measurement of plasma PCNAP1 in HCC patients was found. Area under the curve analysis allowed for a better understanding of the diagnostic value of PCNAP1 for HCC. It is important to note that the onset of HCC is insidious and that patients are often referred to a doctor at an advanced disease stage, which limits treatment options. Therefore, there is an urgent need for an early diagnostic marker with high sensitivity and specificity for improved clinical diagnosis. That need will be the focus of our future work.

## Figures and Tables

**Table 1:** Relationships among plasma levels of PCNAP1 and patient clinicopathologic features.

Clinicopathological parameters	Expression of PCNAP1		*P*	*χ* ^2^
	High	Low		
Gender (*N*)			0.459	6.228
Female	10	10		
Male	46	23		
Age (years)			0.656	5.309
≤60	41	28		
>60	15	5		
TNM stages (*N*)			0.026	10.337
І	9	4		
II	8	9		
III	30	11		
IV	9	9		
Lymph node metastasis (*N*)			0.043	6.718
Yes	49	24		
No	7	9		
Tumor size (cm)			0.037	8.164
≥5	39	17		
<5	17	16		

**Table 2:** ROC curve analysis of the use of plasma PCNAP1 for diagnosis and differential diagnosis of hepatocellular carcinoma.

Characteristics	AUC	SE	*P*	95% CI	Sensitivity	Specificity	Youden index
PCNAP1+AFP (HCC)	0.87	0.02	<0.001	0.82~0.92	78.74%	85.83%	0.65
PCNAP1 (HCC)	0.83	0.03	<0.001	0.78~0.88	70.08%	85.04%	0.55
AFP (HCC)	0.75	0.03	<0.001	0.69~0.82	66.93%	78.74%	0.46
PCNAP1 (colorectal cancer)	0.57	0.04	0.05	0.49~0.65	44.00%	69.29%	0.13
PCNAP1 (gastric cancer)	0.54	0.04	0.35	0.46~0.61	51.00%	60.43%	0.11

## Data Availability

The data used to support the findings of this study are available from the corresponding authors upon request.
